# Supplementary material for: Calcium stabilizes the strongest protein fold
Source: Nat Commun. 2018 Nov 12;9:4764. doi: 10.1038/s41467-018-07145-6 (PMC6232131; doi:10.1038/s41467-018-07145-6)
Supplement: Supplementary file 1 — Supplementary Information [file 41467_2018_7145_MOESM1_ESM.pdf]

Supplementary Information for:

Calcium stabilizes the strongest protein fold

Lukas F. Milles [1], Eduard M. Unterauer [1], Thomas Nicolaus [1], Hermann E. Gaub [1]

1. Lehrstuhl für Angewandte Physik and Center for Nanoscience, Ludwig-Maximilians-University, Amalienstr. 54, 80799 Munich, Germany.

Correspondence and requests for materials should be addressed to H.E.G. (email: gaub@lmu.de) or to L.F.M. (email: lukas.milles@physik.uni-muenchen.de)

Supplementary Figures

S1 Sequence alignment of B domains investigated, including mutants of SdrG B1

S2 Representative force-extension curves for SdrG B1 domain unfolding in 10 mM EDTA and 10 mM Ca^{2+}

S3 Contour length diagram alignments for SdrG B1 domain unfolding in 10 mM EDTA and 10 mM Ca^{2+}

S4 SdrG B1 domain unfolding force distributions in 100 mM citric acid

S5 Mg^{2+} is unable to stabilize SdrG B1 from the weak into the strong state

S6 Detailed unfolding force distributions for the Ca-loop mutants of SdrG B1

S7 Closeups of the Ca^{2+} binding sites of SdrG B1

S8 Bimodal forces of the SdrD B1 strong state

S9 Structural alignment of SdrG B1 and an isopeptide bond-containing pilus domain

S10 Overlapping of unfolding of SdrG B1 and SdrG N2N3:Fgß unbinding forces across varied force loading rates

Supplementary Methods

Protein construct sequences

Spring constants of cantilevers

Supplementary References

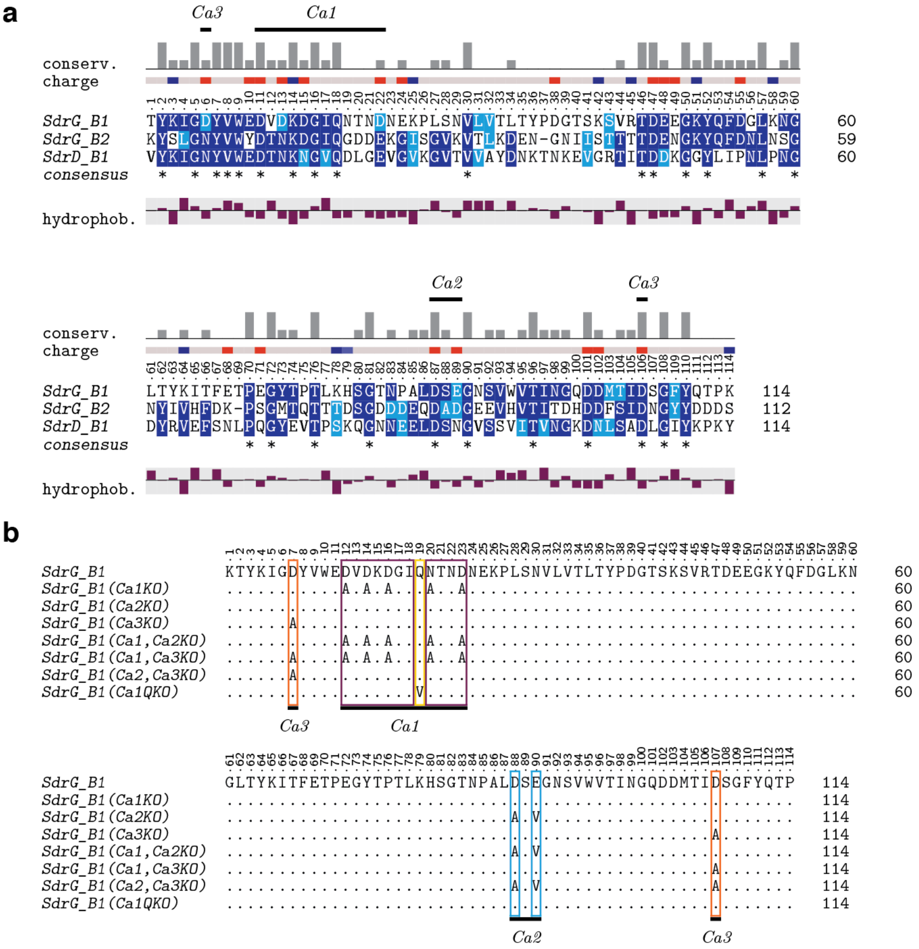

Supplementary Figure 1: Sequence alignment of B domains investigated, including mutants of SdrG B1

(a) Sequence alignment of SdrG B1, SdrG B2, and SdrD B1 with the Ca-binding loops marked. Key residues are conserved or at least similar, e.g. the Aspartic acid at position 107 in the Ca3 loop, and the Glutamine “bridge” between Ca1 and Ca3 in the Ca1 loop.

(b) Mutations introduced in SdrG B1 to isolate Ca^{2+} binding loop function and the glutamine bridge at position 19. Both Figures plotted using TeXShade[1].

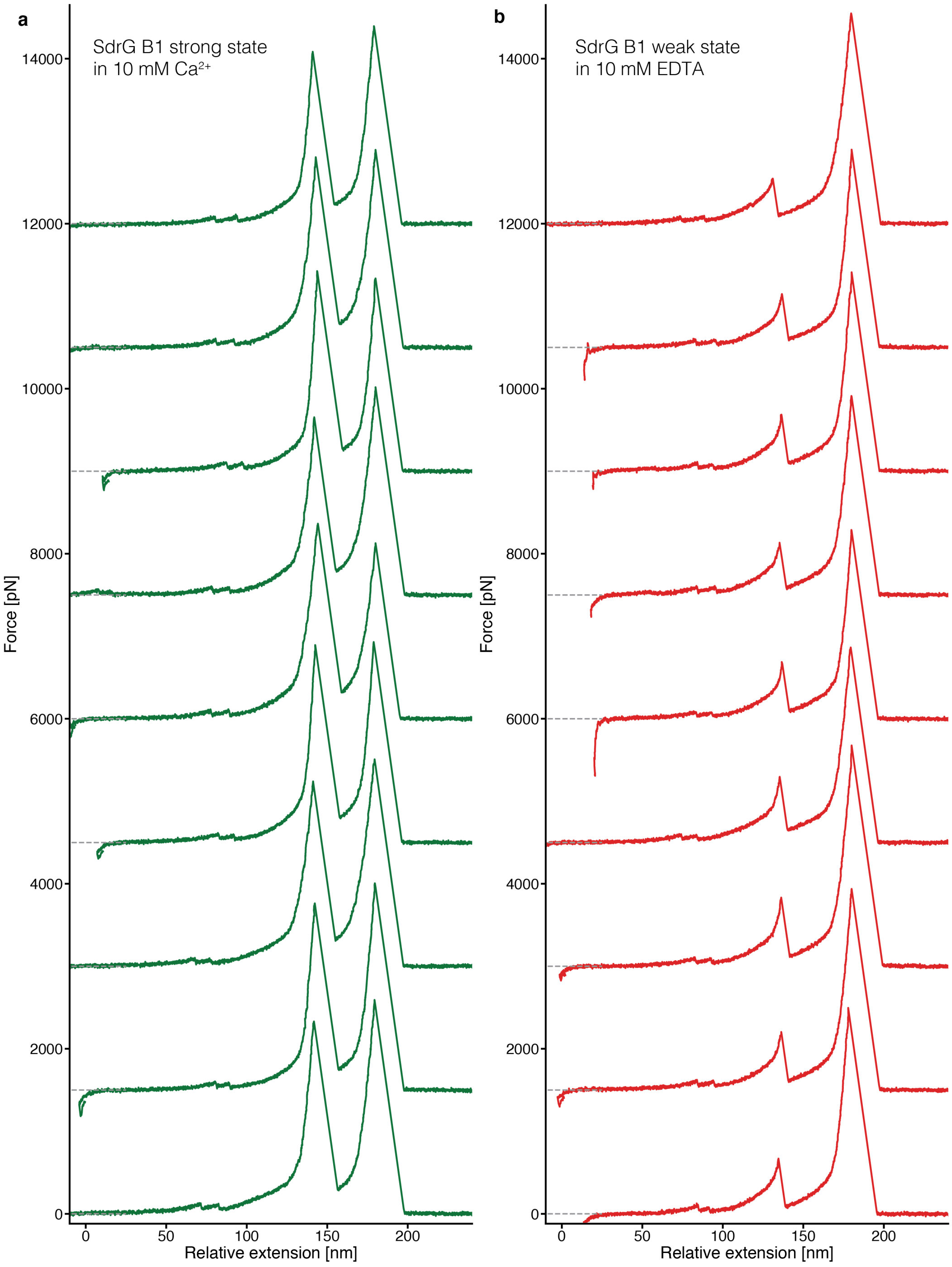

Supplementary Figure 2: Representative force-extension curves for SdrG B1 domain unfolding in 10 mM EDTA and 10 mM Ca^{2+}

Representative force extension traces of SdrG_N2N3-B1 being tethered with Fgß-ddFLN4 at a retraction velocity of 1.6 µm s^{-1}. Curves are each offset by 1500 pN and aligned to the final dissociation event. Zero force or baseline for each curve is shown as gray dashed line near zero extension. Conditions were:

(a) in green, under 10 mM Ca^{2+}. The curves first show the unfolding of the ddFLN4 domain at

around 100 pN with the characteristic intermediate state, visible as a substep. Subsequently the SdrG B1 domain unfolds from the strong state at over 2000 pN. Some traces show the B1 domain unfolding at slightly higher forces than the dissociation event of the SdrG:Fgß interaction, e.g. in the third curve from the top.

(b) in red, under 10 mM EDTA. The curve starts with the ddFLN4 unfolding as above. However, here the SdrG B1 domain unfolds around 650 pN. Receptor-ligand dissociation still occurs within the same force range as above, demonstrating that the B1 domain state has no influence on it.

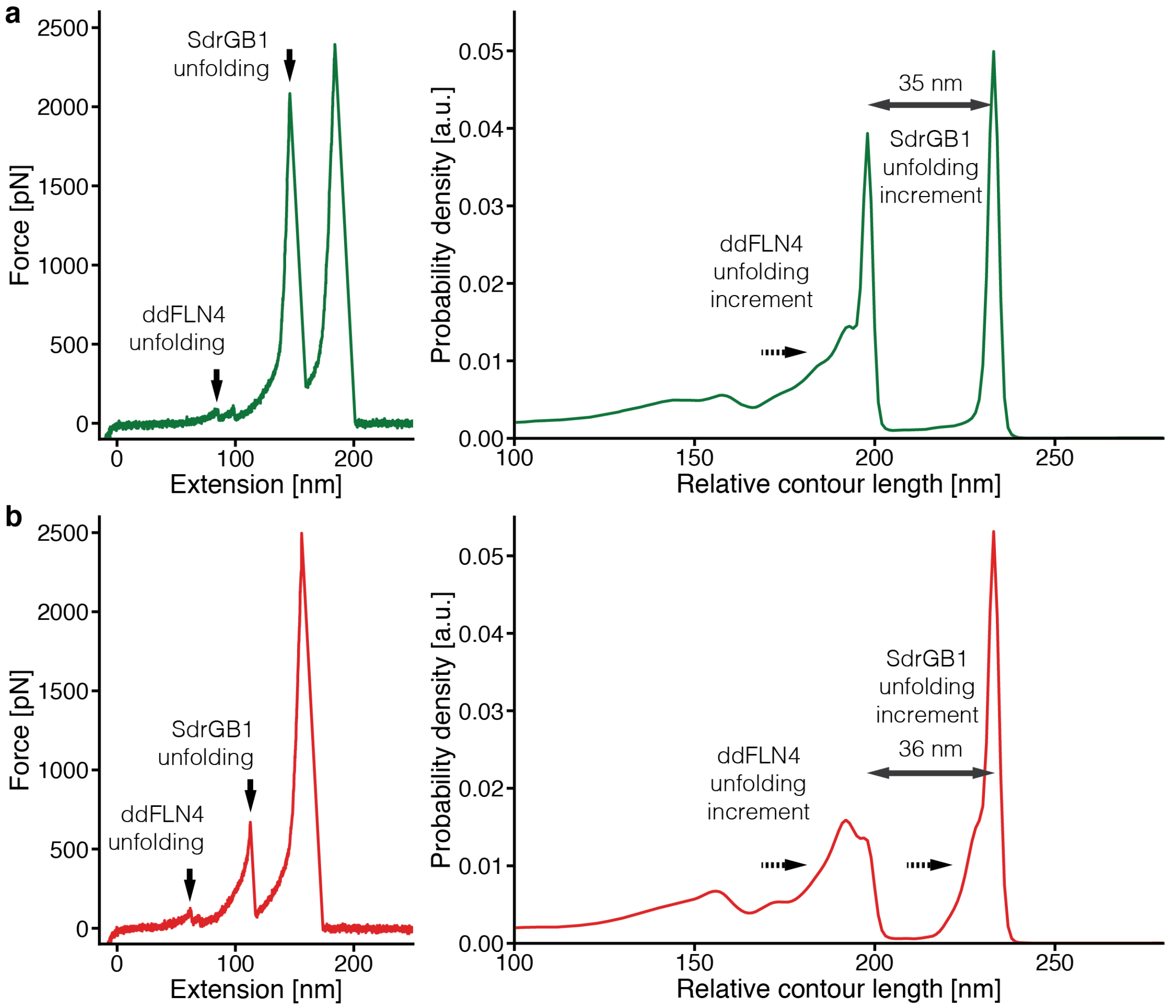

Supplementary Figure 3: Contour length diagram alignments for SdrG B1 domain unfolding in 10 mM EDTA and 10 mM Ca^{2+}

Aligned contour length diagrams (right) with a representative force-distance curve recorded at a retraction velocity of 1.6 µm s^{-1} and assigned unfolding events (left). Contour length diagrams as Gaussian KDEs with a bandwidth of 1 nm assembled for each force-extension curve with the model by Livadaru et al. were aligned to the contour length of the final dissociation event and averaged. The contour length transformations are broader and less defined below 500 pN. This is due to two effects: firstly, a constant persistence length (or bond length for the FRC model) is assumed in the transformation. However due to the differences in persistence length between the PEG linkers and the unfolded protein polypeptide, a mismatch occurs. Secondly, the PEG linker used for surface immobilization undergoes a conformational transition up to around 300 pN, in which it increases its contour length with increasing force. This force-dependent contour length drift is visible in the diagrams, see horizontal, dashed arrows. For a detailed discussion of these effects refer to Ott,

Jobst et al.[2]. As the unfolding events of the SdrG B1 domain occur above 500 pN where the conformational transition of PEG is completed, the largest contour length values, which are incidentally usually the most probable of the transformation, can be used with good confidence. This is particularly important for the weak state. Experimental conditions were:
(a) in green, under 10 mM Ca^{2+}, thus SdrG B1 in the strong state (N = 1754).
(b) in red, under 10 mM EDTA, thus SdrG B1 in the weak state (N = 2269).
Both assembled diagrams show contour length increments (35 nm for the strong state and 36 nm for the weak state) consistent with the expected contour length increment of SdrG B1 of approximately 36 nm and well within the uncertainty of this method as the KDE bandwidth is already 1 nm.

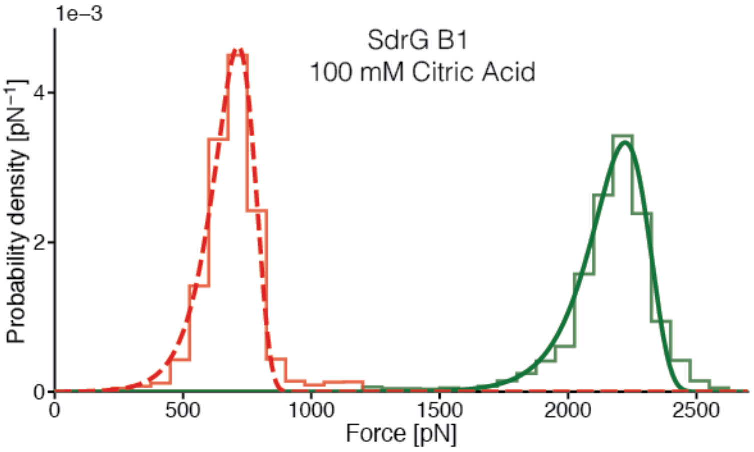

Supplementary Figure 4: SdrG B1 domain unfolding force distributions in 100 mM citric acid

SdrG B1 immobilized on a surface was probed in 100 mM citric acid pH 7.4 at a retraction velocity of 1.6 µm s^{-1}. The majority of domains were in the weak state (59 %, red, N = 2690) and less in the strong state (41%, green, N = 1837). In 10 mM EDTA, a better chelating agent, at similar pH almost all SdrG B1 domains were in the weak state.

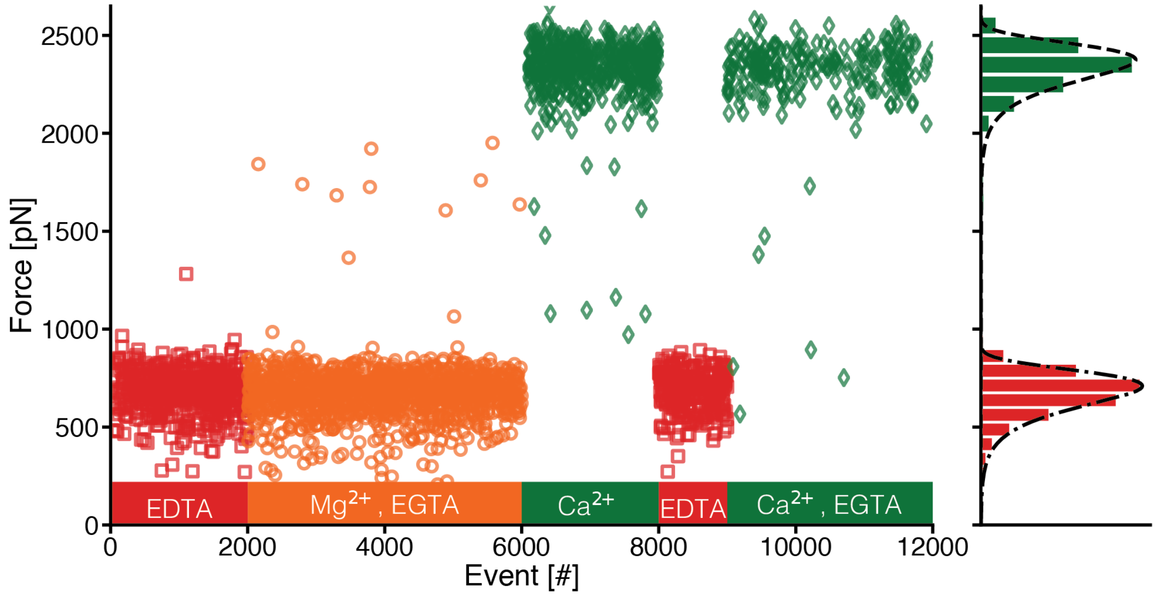

Supplementary Figure 5: Mg^{2+} is unable to stabilize SdrG B1 from the weak into the strong state

SdrG B1 immobilized on a surface was probed at a retraction velocity of 1.6 µm s^{-1} (N = 2796). First in 10 mM EDTA showing exclusively the weak state unfolding. Then in PBS (ultra quality, see methods, pH 7.3) supplemented with 20 mM magnesium acetate (BioXtra, Sigma-Aldrich, St. Louis, MO, USA), and 2 mM EGTA (Carl Roth, Karlsruhe, Germany). The addition of EGTA, which has a much higher affinity for Ca^{2+} than Mg^{2+}, was necessary to remove contaminating Ca^{2+} in the magnesium acetate (containing less than 0.002% calcium according to the manufacturer, which would still compute to on the order of µM Ca^{2+} at 20 mM magnesium acetate – more than enough to switch SdrG B1 into the strong state). As Mg^{2+} was in excess of EGTA, we can assume that at least 18 mM of Mg^{2+} were still freely available in the buffer, however the SdrG B1 weak state remained unchanged. Very few scattered events in the 1000 to 2000 pN range occurred (11 out of 1177), too few to associate them with a clear unfolding pathway, most likely caused by remaining Ca^{2+}, as these events also appear in saturated Ca^{2+} conditions. Thus, we conclude that Mg^{2+} is unable to occupy the Ca^{2+} binding sites in SdrG B1, at least at the 18 mM concentration probed here. Subsequently, the sample was measured in 10 mM Ca^{2+}, recovering the strong state, then 10 mM EDTA to recover the weak state. As a control, to demonstrate that EGTA had no detrimental effect on SdrG B1 domain stability, the sample was finally probed in 10 mM Ca^{2+} supplemented with 2 mM EGTA, in which the strong state reappeared, unchanged.

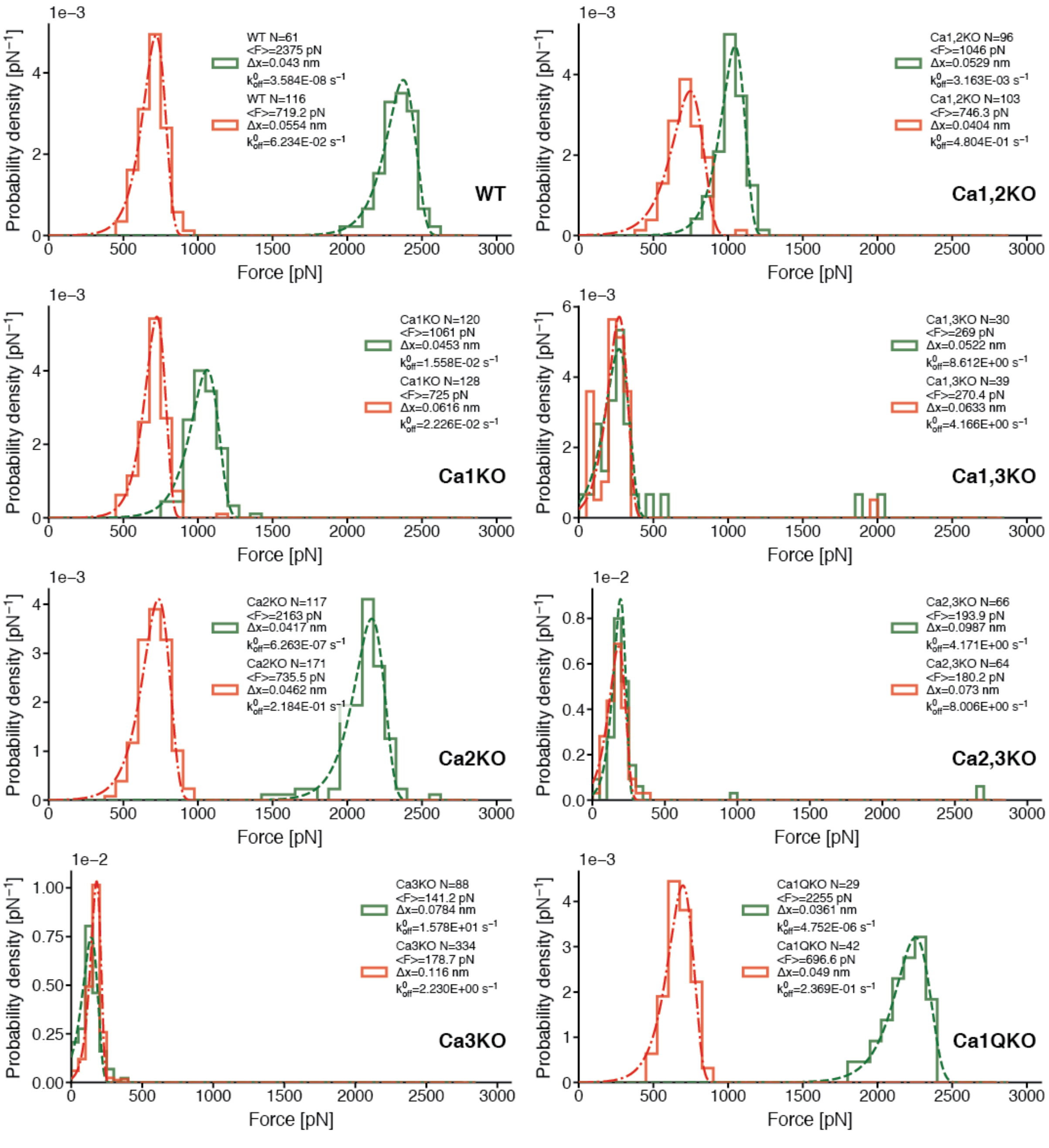

Supplementary Figure 6: Detailed unfolding force distributions for the Ca-loop mutants of SdrG B1.

For each mutant with the functional amino acids replaced, see Fig. S1, (Ca1KO; Ca2KO; Ca3KO) and the double mutants (Ca1,2KO; Ca2,3KO, Ca1,3KO) and the mutant removing the "glutamine bridge" in Ca1 (Ca1QKO) unfolding force distributions are shown. In 10 mM Ca^{2+} shown in red (fits, dashed-dotted line) and 10 mM EDTA in green (fits, dashed line). These distributions were recorded with a single cantilever at a retraction velocity of 1.6 µm s^{-1} with the mutants immobilized in separated spots on a single surface, so all forces can be compared quantitatively. Detailed BE fit parameters and N are shown as inset.

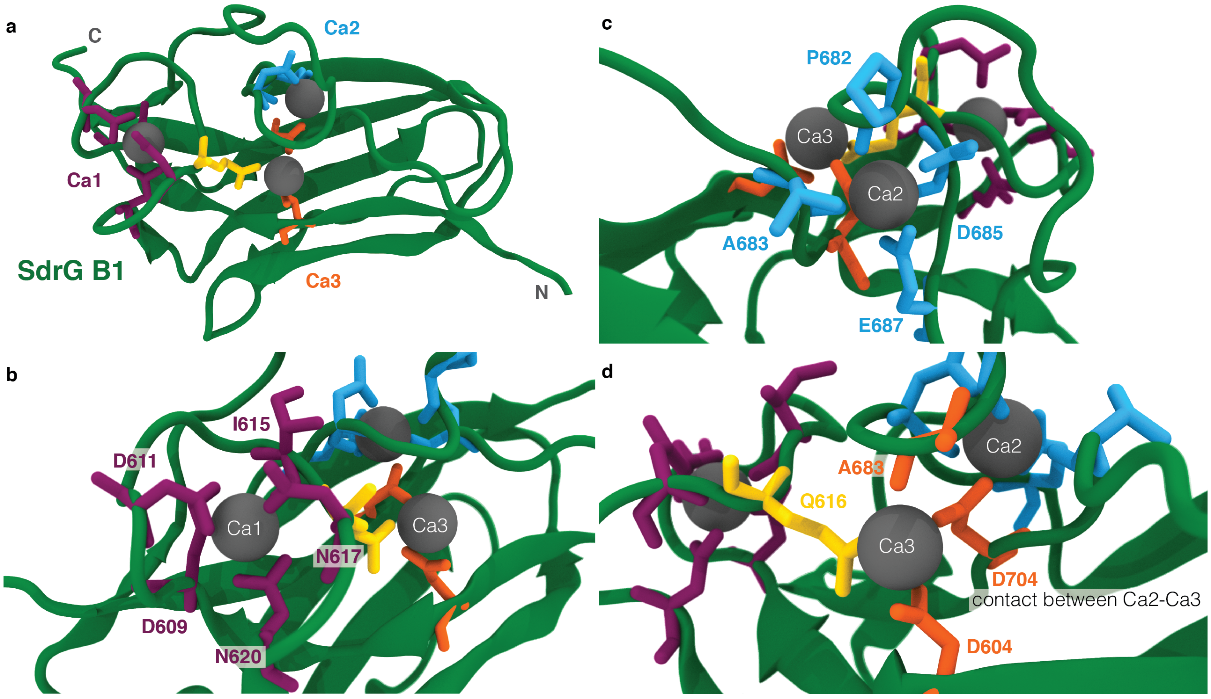

Supplementary Figure 7: Closeups of the Ca^{2+} binding sites of SdrG B1.
(a) Overview of the Ca^{2+} binding sites in the equilibrated SdrG B1 homology model with relevant amino acids in stick representation (Ca1, purple; Ca2 cyan; Ca3 orange, glutamine bridge between Ca1 and Ca3, yellow).
(b) Closeup of the Ca1 site: the backbone oxygen of I615 also contacts Ca1
(c) Closeup of the Ca2 site: backbones of A683 and P682 also contact Ca2, as well as D704, This could explain why the Ca1,2KO mutant (mutating D685A, E687V) was very similar in behavior to the Ca1KO mutant: possibly the remaining amino acids, especially D704 can still coordinate Ca2.
(d) Closeup of the Ca3 site: Q616, the "glutamine bridge" contacts Ca3. D704 also contacts Ca2. A backbone oxygen in A683 on the Ca2 binding loop also coordinates Ca3.

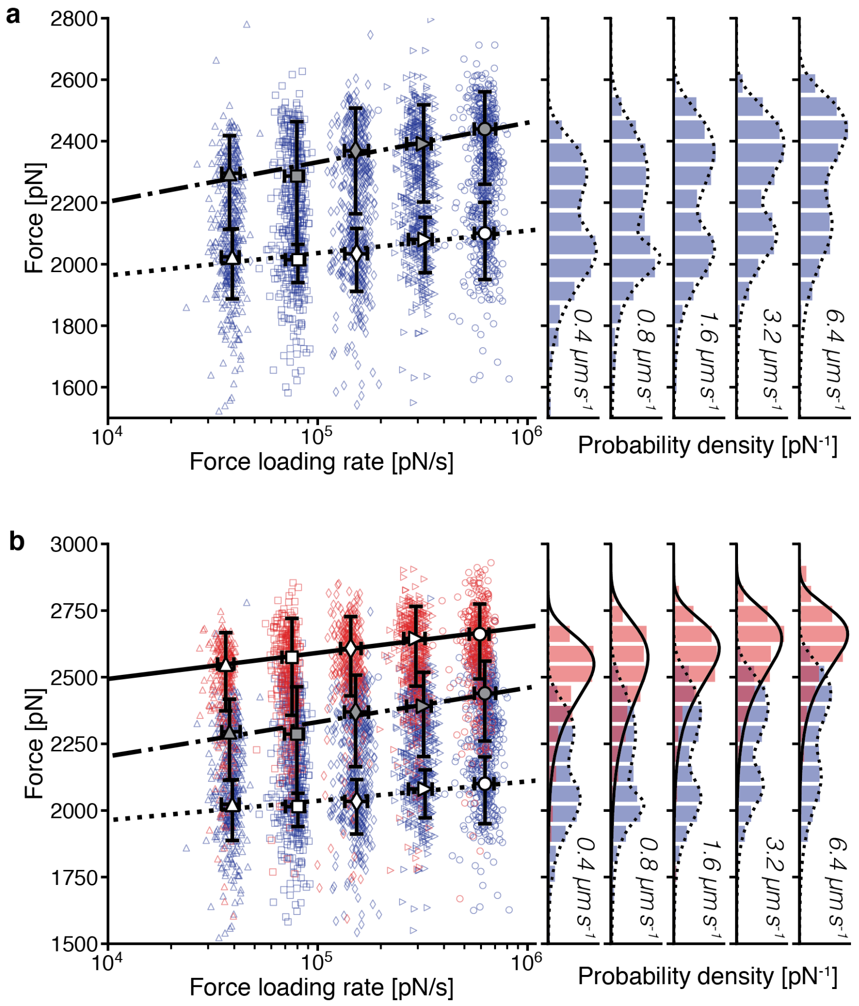

Supplementary Figure 8: Bimodal forces of the SdrD B1 strong state.

(a) Unfolding force distributions of SdrD B1 in 25 nM Ca^{2+} with bimodal unfolding events a weaker event around 2050 pN and a strongest event around 2300 pN, shown in blue, fit with two superimposed BE functions: 0.4 µm s^{-1} (triangles, N = 434), 0.8 µm s^{-1} (squares, N = 505), 1.6 µm s^{-1} (diamonds, N = 516), 3.2 µm s^{-1} (forward triangles, N = 596), 6.4 µm s^{-1} (circles, N = 606), BE fits (weaker state: open, white symbols and dashed line, Δx = 0.13 nm, k_{off}^0 = 3.4E-25 s^{-1}, strongest state: grey, open symbols and dash-dotted line, Δx = 0.074 nm, k_{off}^0 = 1.3E-15 s^{-1}). Notably, the weaker state has a very flat slope, reflected in the extremely low k_{off}^0.

(b) Data from (a) overlaid with the ClfB:DK handle unbinding forces in red: 0.4 µm s^{-1} (triangles, N = 296), 0.8 µm s^{-1} (squares, N = 354), 1.6 µm s^{-1} (diamonds, N = 345), 3.2 µm s^{-1} (forward triangles, N = 422), 6.4 µm s^{-1} (circles, N = 431), BE fit (continuous line and open markers, Δx = 0.098 nm, k_{off}^0 = 7.2E-24 s^{-1}). The less steep force loading rate dependency of the ClfB:DK receptor ligand rupture force compared to the SdrD B1 strongest state unfolding induces a fingerprint bias[3] that becomes more relevant at higher force loading rates.

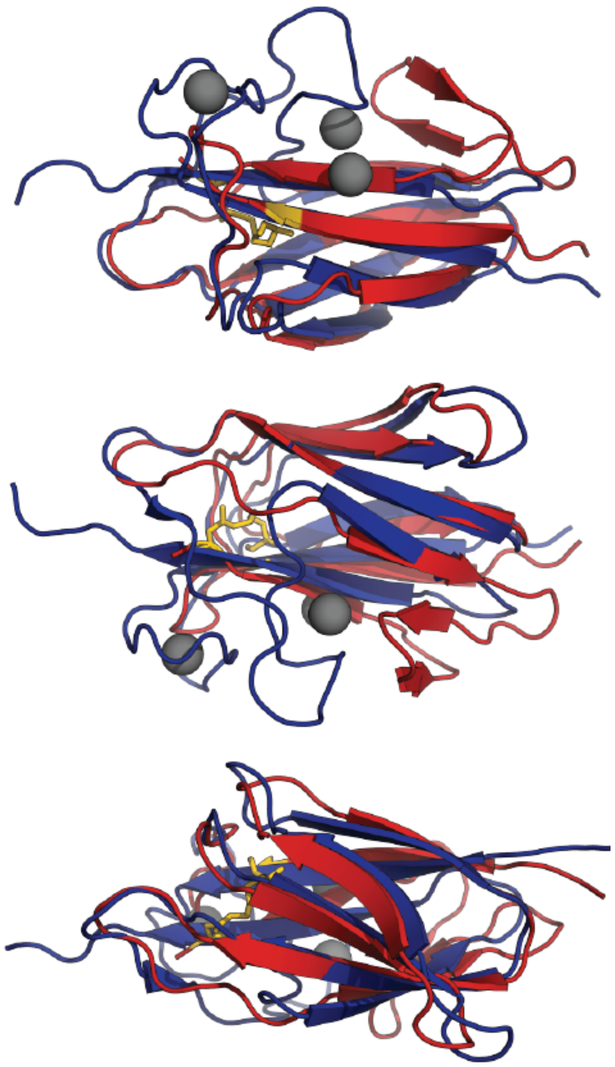

Supplementary Figure 9: Structural alignment of SdrG B1 and an isopeptide bond-containing pilus domain.
SdrD B1 crystal structure (PDB 4JDZ[4], blue, Ca^{2+} ions coordinated as grey spheres) in structural alignment with Fibronectin binding protein fba2 from *S. pyogenes* (red, PDB 2X5P[5], UniProt: Q8G9G1) The isopeptide bond connecting the N- and C-terminal ß-sheets in is highlighted in yellow stick representation, the domains are shown in different perspectives. The underlying ß-sandwich fold aligns well, major differences are the missing Ca^{2+} binding loops in fba2, which figuratively lie on top of the SdrG B1 fold and close it. In fba2 instead the isopeptide bond locks N- and C-terminal ß-sheet.

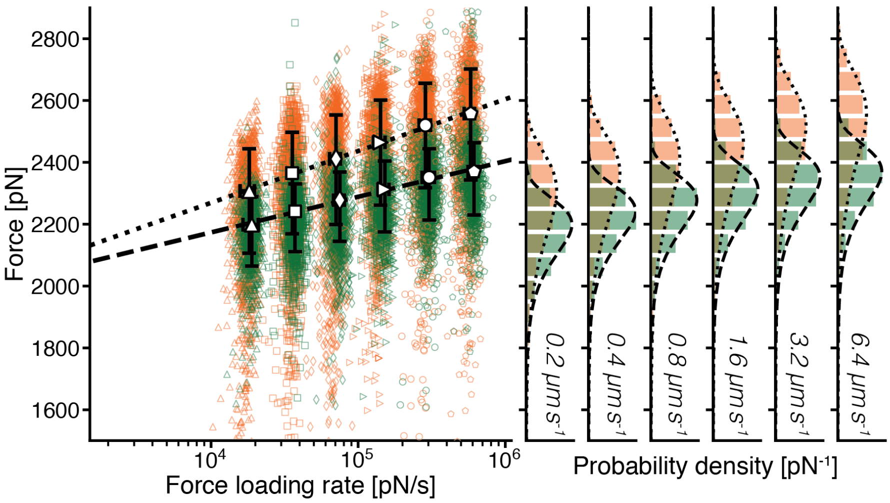

Supplementary Figure 10: Overlapping of unfolding of SdrG B1 and SdrG N2N3:Fgß unbinding forces across varied force loading rates.

Dynamic force spectra of:

SdrG N2N3:Fgß unbinding in orange: 0.2 µm s^{-1} (triangles, N = 1588), 0.4 µm s^{-1} (squares, N = 1958), 0.8 µm s^{-1} (diamonds, N = 2142), 1.6 µm s^{-1} (forward triangles, N = 2144), 3.2 µm s^{-1} (circles, N = 1909), 6.4 µm s^{-1} (pentagons, N = 2133), BE fit (dotted line, Δx = 0.057 nm, k_{off}^0 = 3.9E-12 s^{-1})

SdrG B1 unfolding in strong state in green: 0.2 µm s^{-1} (triangles, N = 848), 0.4 µm s^{-1} (squares, N = 1130), 0.8 µm s^{-1} (diamonds, N = 1164), 1.6 µm s^{-1} (forward triangles, N = 1209), 3.2 µm s^{-1} (circles, N = 1044), 6.4 µm s^{-1} (pentagons, N = 1146), BE fit (dashed line, Δx = 0.083 nm, k_{off}^0 = 2.8E-17 s^{-1})

At loading rates around 10^4 pN/s a strong overlap of receptor ligand handle unbinding and SdrG B1 unfolding, resulting in a fingerprint bias. The steeper dependency of the rupture force on the force loading rate of SdrG N2N:Fgß dissociation compared to the flatter slope of SdrG B1 unfolding, alleviates this effect at higher force loading rates of larger than 10^5 pN/s. A load dissipater function of the SdrG B1 domain at such rates is possible.

Supplementary Methods

Protein construct sequences

All constructs were cloned onto pET28a vectors and contain a 6xHIS (HHHHHH) tag for purification and a ybbr-tag (DSLEFIASKLA) for covalent surface anchoring. Sequences may contain a HRV 3C Protease cleavage site (LEVLFQGP) or a sortase motif for covalent surface anchoring (C-terminus: LPETGG, N-terminus: MGGG), which were not used here. The wild-type ddFLN4 fingerprint contains a cysteine that has been mutated as C18S to avoid a potential cross-reaction to Maleimides.

SdrG (N2_N3 domains) – 6xHIS – ybbr (Adgene ID: 101238)

MGTEQGSNVNHLIKVTDQSITEGYDDSDGIIKAHDAENLIYDVTFEVDDKVKSGDTMTVNIDKNTVPSDLTD
SFAIPKIKDNSGEIIATGTYDNTNKQITYTFTDYVDKYENIKAHLKLTSYIDKSKVPNNNTKLDVEYKTALS
SVNKTITVEYQKPNENRTANLQSMFTNIDTKNHTVEQTIYINPLRYSAKETNVNISGNGDEGSTIIDDSTII
KVYKVGDNQNLPDSNRIYDYSEYEDVTNDDYAQLGNNNDVNINFGNIDSPYIIKVISKYDPNKDDYTTIQQT
VTMQTTINEYTGEFRTASYDNTIAFSTSSGQGQGDLPPEKT
ELKLPRSRHHHHHHGSLEVLFQGPDSLEFIASKLA

SdrG (N2_N3 domains – B1 – B2)– 6xHIS – ybbr (Addgene ID: 117979)

MGTEQGSNVNHLIKVTDQSITEGYDDSDGIIKAHDAENLIYDVTFEVDDKVKSGDTMTVNIDKNTVPSDLTD
SFAIPKIKDNSGEIIATGTYDNTNKQITYTFTDYVDKYENIKAHLKLTSYIDKSKVPNNNTKLDVEYKTALS
SVNKTITVEYQKPNENRTANLQSMFTNIDTKNHTVEQTIYINPLRYSAKETNVNISGNGDEGSTIIDDSTII
KVYKVGDNQNLPDSNRIYDYSEYEDVTNDDYAQLGNNNDVNINFGNIDSPYIIKVISKYDPNKDDYTTIQQT
VTMQTTINEYTGEFRTASYDNTIAFSTSSGQGQGDLPPE
KTYKIGDYVWEDVDKDGIQNTNDNEKPLSNVLVTLTYPDGTSKSVRTDEEGKYQFDGLKNGLTYKITFETPE
GYTPTLKHSGTNPALDSEGNSVWVTINGQDDMTIDSGFYQTP
KYSLGNYVWYDTNKDGIQGDDEKGISGVKVTLKDENGNIISTTTTDENGKYQFDNLNSGNYIVHFDKPSGMT
QTTTDSGDDDEQDADGEEVHVTITDHDDFSIDNGYYDDDS
ELKLPRSRHHHHHHGSLEVLFQGPDSLEFIASKLA

SdrG (N2_N3 domains – B1)– 6xHIS – ybbr (Addgene ID: 117980)

MGTEQGSNVNHLIKVTDQSITEGYDDSDGIIKAHDAENLIYDVTFEVDDKVKSGDTMTVNIDKNTVPSDLTD
SFAIPKIKDNSGEIIATGTYDNTNKQITYTFTDYVDKYENIKAHLKLTSYIDKSKVPNNNTKLDVEYKTALS
SVNKTITVEYQKPNENRTANLQSMFTNIDTKNHTVEQTIYINPLRYSAKETNVNISGNGDEGSTIIDDSTII
KVYKVGDNQNLPDSNRIYDYSEYEDVTNDDYAQLGNNNDVNINFGNIDSPYIIKVISKYDPNKDDYTTIQQT
VTMQTTINEYTGEFRTASYDNTIAFSTSSGQGQGDLPPE
KTYKIGDYVWEDVDKDGIQNTNDNEKPLSNVLVTLTYPDGTSKSVRTDEEGKYQFDGLKNGLTYKITFETPE
GYTPTLKHSGTNPALDSEGNSVWVTINGQDDMTIDSGFYQTP
ELKLPRSRHHHHHHGSLEVLFQGPDSLEFIASKLA

ClfB (N2_N3 domains) – 6xHIS – ybbr (Addgene ID: 101717)

MGTPVVNAADAKGTNVNDKVTASNFKLEKTTFDPNQSGNTFMAANFTVTDKVKSGDYFTAKLPDSLTGNGDV
DYSNSNNTMPIADIKSTNGDVVAKATYDILTKTYTFVFTDYVNNKENINGQFSLPLFTDRAKAPKSGTYDAN
INIADEMFNNKITYNYSSPIAGIDKPNGANISSQIIGVDTASGQNTYKQTVFVNPKQRVLGNTWVYIKGYQD
KIEESSGKVSATDTKLRIFEVNDTSKLSDSYYADPNDSNLKEVTDQFKNRIYYEHPNVASIKFGDITKTYVV

LVEGHYDNTGKNLKTQVIQENVDPVTNRDYSIFGWNNENVVRYGGGSADGDSAV
ELKLPRSRHHHHHHGSLEVLFQGPDSLEFIASKLA

Fgß – linker – ddFLN4(C18S) – 6xHIS – ybbr (Addgene ID: 101239)

MGTNEEGFFSARGHRPLDGSGSGSGSAGTGSG
ADPEKSYAEGPGLDGGESFQPSKFKIHAVDPDGVHRTDGGDGFVVTIEGPAPVDPVMVDNGDGTYDVEFEPK
EAGDYVINLTLDGDNVNGFPKTVTVKPAP
SGHHHHHHGSDSLEFIASKLA

ybbr – 6xHIS – ddFLN4(C18S) – linker – DK

MDSLEFIASKLAHHHHHHGS
ADPEKSYAEGPGLDGGESFQPSKFKIHAVDPDGVHRTDGGDGFVVTIEGPAPVDPVMVDNGDGTYDVEFEPK
EAGDYVINLTLDGDNVNGFPKTVTVKPAP
GSGSGSGSQSGSSGSGSNGD

MGGG – ybbr – 6xHIS – SdrG_B1 – linker – DK (Addgene ID: 117981)

MGGGDSLEFIASKLAHHHHHHGSAPE
KTYKIGDYVWEDVDKDGIQNTNDNEKPLSNVLVTLTYPDGTSKSVRTDEEGKYQFDGLKNGLTYKITFETPE
GYTPTLKHSGTNPALDSEGNSVWVTINGQDDMTIDSGFYQTP
GSGSQSGSSGSGSNGD

MGGG – ybbr – 6xHIS – [SdrG_B1 mutant] – linker – DK

These constructs are identical except for the mutations in SdrG_B1, see sequences as inserts below. Ca^{2+} coordinating residues were mutated to Alanines or Valines.

MGGGDSLEFIASKLAHHHHHHGSAPE
[>SdrG_B1 mutant]
GSGSQSGSSGSGSNGD

>SdrG_B1(Ca1KO)

KTYKIGDYVWEAVAKAGIQATNANEKPLSNVLVTLTYPDGTSKSVRTDEEGKYQFDGLKNGLTYKITFETPE
GYTPTLKHSGTNPALDSEGNSVWVTINGQDDMTIDSGFYQTP

>SdrG_B1(Ca2KO)

KTYKIGDYVWEDVDKDGIQNTNDNEKPLSNVLVTLTYPDGTSKSVRTDEEGKYQFDGLKNGLTYKITFETPE
GYTPTLKHSGTNPALASVGNSVWVTINGQDDMTIDSGFYQTP

>SdrG_B1(Ca3KO)

KTYKIGAYVWEDVDKDGIQNTNDNEKPLSNVLVTLTYPDGTSKSVRTDEEGKYQFDGLKNGLTYKITFETPE
GYTPTLKHSGTNPALDSEGNSVWVTINGQDDMTIASGFYQTP

>SdrG_B1(Ca1,Ca2KO)

KTYKIGDYVWEAVAKAGIQATNANEKPLSNVLVTLTYPDGTSKSVRTDEEGKYQFDGLKNGLTYKITFETPE
GYTPTLKHSGTNPALASVGNSVWVTINGQDDMTIDSGFYQTP

>SdrG_B1(Ca1,Ca3KO)

KTYKIGAYVWEAVAKAGIQATNANEKPLSNVLVTLTYPDGTSKSVRTDEEGKYQFDGLKNGLTYKITFETPE
GYTPTLKHSGTNPALDSEGNSVWVTINGQDDMTIASGFYQTP

>SdrG_B1(Ca2,Ca3KO)

KTYKIGAYVWEDVDKDGIQNTNDNEKPLSNVLVTLTYPDGTSKSVRTDEEGKYQFDGLKNGLTYKITFETPE
GYTPTLKHSGTNPALASVGNSVWVTINGQDDMTIASGFYQTP

>SdrG_B1(Ca1QKO)

KTYKIGDYVWEDVDKDGIVNTNDNEKPLSNVLVTLTYPDGTSKSVRTDEEGKYQFDGLKNGLTYKITFETPE
GYTPTLKHSGTNPALDSEGNSVWVTINGQDDMTIDSGFYQTP

MGGG – ybbr – 6xHIS – SdrG_B2 – linker – DK (Addgene ID: 117982)

MGGGDSLEFIASKLAHHHHHHGSA
KYSLGNYVWYDTNKDGIQGDDEKGISGVKVTLKDENGNIISTTTTDENGKYQFDNLNSGNYIVHFDKPSGMT
QTTTDSGDDDEQDADGEEVHVTITDHDDFSIDNGYYDDD
SGSGSQSGSSGSGSNGD

MGGG – ybbr – 6xHIS – SdrD_B1 – linker – DK (Addgene ID: 117983)

MGGGDSLEFIASKLAHHHHHHGSASGGAGQE
VYKIGNYVWEDTNKNGVQDLGEVGVKGVTVVAYDNKTNKEVGRTITDDKGGYLIPNLPNGDYRVEFSNLPQG
YEVTPSKQGNNEELDSNGVSSVITVNGKDNLSADLGIYKP
KYNLGDYVGSGSQSGSSGSGSNGD

Spring constants of cantilevers

All measurements were conducted with BioLever Mini AC40TS (Olympus, Tokyo, Japan) cantilevers. The uncertainty of each value is approximately 10%[6], making quantitative force comparisons between measurements challenging. When absolute comparisons were needed data were recorded with a single cantilever, e.g. in Fig. 3 c,d

Figure 1	e, f	$k_{Cantilever}$ = 156 pN/nm
Figure 2	a, b	$k_{Cantilever}$ = 141 pN/nm
	c	$k_{Cantilever}$ = 145 pN/nm
	d	$k_{Cantilever}$ = 74 pN/nm
Figure 3	c, d	$k_{Cantilever}$ = 140 pN/nm
Figure 4	c	$k_{Cantilever}$ = 147 pN/nm
	d, e	$k_{Cantilever}$ = 133 pN/nm
Figure S2		(same as in Fig. 2 a,b)
Figure S3		(same as in Fig. 2 a,b)
Figure S4		$k_{Cantilever}$ = 133 pN/nm
Figure S5		$k_{Cantilever}$ = 158 pN/nm
Figure S6		(same as in Fig. 3 c, d)
Figure S8		$k_{Cantilever}$ = 139 pN/nm
Figure S10		(same as in Fig. 2 c)

Supplementary References

1. Beitz, E. TeXshade: shading and labeling of multiple sequence alignments using LaTeX2e. *Bioinformatics* **16,** 135–139 (2000).
2. Ott, W. *et al.* Elastin-like Polypeptide Linkers for Single-Molecule Force Spectroscopy. *ACS Nano* acsnano.7b02694 (2017). doi:10.1021/acsnano.7b02694
3. Schoeler, C., Verdorfer, T., Gaub, H. E. & Nash, M. A. Biasing effects of receptor-ligand complexes on protein-unfolding statistics. *Phys. Rev. E* **94,** 042412 (2016).
4. Wang, X., Ge, J., Liu, B., Hu, Y. & Yang, M. Structures of SdrD from Staphylococcus aureus reveal the molecular mechanism of how the cell surface receptors recognize their ligands. *Protein Cell* **4,** 277–285 (2013).
5. Oke, M. *et al.* The scottish structural proteomics facility: Targets, methods and outputs. *J. Struct. Funct. Genomics* **11,** 167–180 (2010).
6. Brand, U. *et al.* Comparing AFM cantilever stiffness measured using the thermal vibration and the improved thermal vibration methods with that of an SI traceable method based on MEMS. *Meas. Sci. Technol.* **28,** 034010 (2017).
